# Inhibition of pathological retinal neovascularization by semaphorin 3A

**Published:** 2013-06-27

**Authors:** Wenzhen Yu, Yujing Bai, Na Han, Fei Wang, Min Zhao, Lvzhen Huang, Xiaoxin Li

**Affiliations:** 1 Key Laboratory of Vision Loss and Restoration, Ministry of Education, Department of Ophthalmology, Peking University People’s Hospital, Beijing, China; 2 Department of Orthopaedics and Trauma, Peking University People's Hospital, Beijing, China

## Abstract

**Objective:**

Pathological retinal angiogenesis is a major cause of vision loss. Semaphorin 3A (Sema3A), a chemorepellent guidance protein, plays crucial roles in neural and vascular patterning. To identify its role in retinal neovascularization, we investigated its antiangiogenic effects.

**Methods:**

Human umbilical vein endothelial cells (HUVECs) were used for the in vitro study, and an oxygen-induced retinopathy (OIR) mouse model was used for the in vivo study. The HUVECs were incubated with Sema3A, and cell proliferation, migration, apoptosis, cell cycle, tube formation, and c-Jun N-terminal kinase (JNK) and p38 mitogen-activated protein kinases (p38 MAPK) signaling pathways were measured using Cell Counting Kit-8, Transwell, flow cytometry, Matrigel assays, and western blot. C57BL/6J mouse pups were exposed to 75% oxygen for 5 days and then brought to room air and injected with Sema3A intravitreously. At postnatal day 18, the retinal nonperfused areas were measured. The in vitro and in vivo vascular endothelial growth factor-165 (VEGF_165_) secretion was measured using enzyme-linked immunosorbent assay.

**Results:**

Sema3A not only inhibited VEGF_165_-induced proliferation, but also induced cell cycle arrest in a dose-dependent manner. Furthermore, Sema3A inhibited migration and tube formation, both in general and in VEGF_165_-containing culture medium. Using an enzyme-linked immunosorbent assay, we showed that Sema3A did not affect VEGF_165_ secretion, but it did impede VEGF_165_ function. Additionally, Sema3A significantly inhibited the phosphorylation of the JNK and p38MAPK signaling pathways. When administered intravitreously, Sema3A reduced the pathological vascular changes seen in the retinal neovascularization OIR model.

**Conclusions:**

These results suggest that the administration of Sema3A could be a useful therapeutic strategy for preventing hypoxia/ischemic-induced retinal neovascularization.

## Introduction

Pathological retinal angiogenesis is a major cause of vision loss in various diseases, including retinopathy of prematurity (ROP), diabetic retinopathy, and age-related macular degeneration (AMD) [1,2]. Vascular endothelial growth factor (VEGF) and its receptors have been demonstrated to play major roles in the generation and progression of neovascular eye diseases; they have therefore become the ideal targets for antiangiogenesis therapy [3]. However, anti-VEGF agents can induce local and systemic side effects. It has been shown that the systematic use of anti-VEGF drugs will induce hypertension because of vascular contraction [1], and intravitreous injection will result in the contraction of the retina’s proliferative membranes due to the action of the fibroblast cells and other components in the membranes, which could cause retinal holes [4]. Thus, the exploration and evaluation of new antineovascularization compounds is greatly needed.

Semaphorin 3A (Sema3A) is an endogenous secreted protein that belongs to the class 3 semaphorin family (Sema3), which were originally identified as axonal guidance molecules and were also implicated in vessel pathfinding and network formation [5]. Neuropilin 1 and 2 (Nrp1 and Nrp2) and the type A/D plexins (Plxns) act as the ligands binding and the signal transducing subunits of the Sema3 receptor complexes on the surface of endothelial cells (ECs) [5]. As a special member of the Sema3 family, Sema3A binds to Nrp1 exclusively at first and then combines with PlexinA1–4 as a complex (Nrp1/PlexA1–4). In this receptor complex, Nrp1 acts as a binding element, while PlexA1–4 acts as a signal-transducing element [6]. Since the discovery of Sema3A, a variety of studies have reported its effects on neuronal cell migration, tumor metastasis, and vascular genesis [7-9]. The effects of Sema3A on retina pathological neovascularization, however, have not been documented. In the present study, we extensively investigated the antiangiogenic effects and possible mechanisms of Sema3A in retinal neovascularization for the first time. The encouraging results of our study provide a useful therapeutic strategy for the treatment of retinal neovascularization.

## Methods

### Cells and animals

Human umbilical vein endothelial cells (HUVECs, American type culture collection (ATCC), CRL-1730) were cultured as previously described [10]. HUVECs were cultured in 10% fetal bovine serum containing culture medium as ATCC recommended. Neonatal mice (C57BL/6J) were obtained from the animal center of Peking University and were raised in the animal room of the Peking University People’s Hospital. This study adhered to the Association for Research in Vision and Ophthalmology Statement for the Use of Animals in Ophthalmic and Vision Research and was performed in accordance with the guidelines provided by the Animal Care Use Committee of Peking University. The animals were housed with free access to laboratory food and water and were kept in a 12h:12h light-dark cycle.

### Proliferation assays and vascular endothelial growth factor-165 measurement by enzyme-linked immunosorbent assay in human umbilical vein endothelial cells

Sema3A (Sino Biologic Inc., 50,631-M01H) was incubated with HUVECs in 96-well plates for 24, 48, and 72 h at concentrations of 250 ng/ml and 500 ng/ml in either general culture medium (10% fetal bovine serum [FBS]) or VEGF_165_ (25 ng/ml, R&D, 293-VE-containing medium). Cell Counting Kit-8 (Dojindo, Shanghai) assays were performed according to the manufacturer’s instructions. Briefly, after adding 10 ml of CCK-8 to each well, the cells were incubated at 37 °C for another 30–60 min. Absorbance was measured with an enzyme-linked immunosorbent assay (ELISA) plate reader at a wavelength of 450 nm. Each experiment was repeated in five wells and was duplicated at least three times. With the same treatment process, after incubation times of 24, 48, and 72 h, the cell culture supernatant was harvested and centrifuged. Free VEGF_165_ protein in the culture medium was measured by an ELISA kit (Bostar, EK0575) according to the manufacturer’s instructions.

### Migration assay

HUVECs migration was assayed by Transwell (Corning, US, Cat#3422) as described previously [10]. Briefly, 2×10^4^ cells were placed in the top part of a serum-free medium. Dulbecco's Modified Eagle Medium (DMEM, Hyclone, Grand Island, NY; containing 10% FBS) with 250 ng/ml and 500 ng/ml Sema3A or Sema3A with VEGF_165_ (25 ng/ml) was placed in the bottom chamber. All migration assays were conducted at 37 °C for 5 h, and then the cells were fixed with 4% paraformaldehyde (PFA) and stained with 4',6-diamidine-2'-phenylindole dihydrochloride (DAPI, Roche, Germany, Cat# 236276). The cells that had not migrated were removed with a cotton swab, and the membrane was imaged with fluorescence microscopy (Zeiss Axiophot, Thornwood, NY). Cells from five random view fields were counted, and the average was used for statistical analysis.

### Flow cytometry analysis of human umbilical vein endothelial cell apoptosis and the cell cycle

HUVECs apoptosis study (FITC Annexin V Apoptosis Detection Kit; BD Science) and cell cycle analysis (Cycletest Plus DNA Reagent Kit; BD Science) were performed according to the manufacturer’s instructions and as previously reported [10]. Briefly, the HUVECs (1×10^6^) were seeded in six-well plates and incubated for 24, 48, and 72 h with Sema3A or VEGF_165_ plus Sema3A, or were used as controls. The samples were analyzed by flow cytometer (FACSCalibur; BD Biosciences, Franklin Lakes, NJ), and the experiments were performed in triplicate and repeated three times.

### Tube formation study

According to the manufacturer’s instructions and our previous report [10], 150 μl of Matrigel (BD Sciences, Cat#354234) solution was poured into 48-well plates and was then incubated at 37 °C for 30 min. The HUVECs (5×10^4^ per well) were treated with Sema3A or Sema3A plus VEGF_165_ or were used as controls, and then the cells were seeded on the Matrigel and cultured for 8–10 h. The networks in the Matrigel from five randomly chosen fields were counted and photographed. The length of the tube was measured by Image J software. The experiments were repeated three times.

### Western blot analysis

The HUVECs were prepared with protein extraction and protease inhibitor kits (Pierce). After centrifugation, the supernatant was collected, and the protein lysate was measured with a BCA protein assay kit (Pierce) according to the manufacturer’s instructions. Briefly, diluted albumin standards were prepared for calculate the formula. 25 ml unknown samples were loaded into 96-well plate in three repeated wells. After adding 200 ml working agent into the wells, mix plate thoroughly on a plate shaker for 30 s. Cover plate and incubate at 37°C for 30 min, and measure the absorbance at 560 nm on a plate reader. Equal amounts of protein were loaded and analyzed by immunoblotting. The proteins were visualized with enhanced chemiluminescence western blot detection reagents (Pierce). The band densities of c-Jun N-terminal kinase (JNK, CST, Cat#9258), phosphorylation-c-Jun N-terminal kinase (p-JNK, CST, Cat#4668), p38 mitogen-activated protein kinases (p38 MAPK, CST, Cat#8690), and phosphorylation-p38 MAPK (p-p38 MAPK, CST, Cat#4511) were tested. Western blot analysis was repeated three times, and qualitatively similar results were obtained.

### Induction of an oxygen-induced retinopathy mouse model and assessment of the nonperfusion area

C57BL/6J pups were exposed to hyperoxia (75% oxygen) for five days, from postnatal day 7 (P7) to P12, and were brought back to normoxia from P12 to P17. At P12, the oxygen-induced retinopathy (OIR) mice were injected with Sema3A (1.5 μl, 100 ng/ml, 250 ng/ml) or immunoglobulin G (IgG) intravitreously (1.5 μl, 250 ng/ml). At P18, the mice were sacrificed and were then perfused with 0.5 ml of PBS containing 50 mg of 2×10^6^ MW fluorescein–dextran–fluorescein isothiocyanate (Sigma, St. Louis, MO). After eye fixation, the retinas were flat mounted and photographed. The nonperfused areas (NPA) were analyzed with Image J software. The ratio of the NPA compared to the whole retina was determined.

### Retinal vascular endothelial growth factor-165 concentration measurement

After injections with Sema3A or control for 5 days, the C57 pups were sacrificed at P18, and the retinas were separated as stated in the western blot analysis section. VEGF_165_ (pg/ml protein) in the clarified supernatant was measured with an ELISA kit (Bostar, EK0541). All of the experiments for the ELISA test were performed in five pups, and each experiment was repeated three times.

### Statistical analysis

The data analysis was performed using the statistical software Prism 5 (GraphPad Software Inc., San Diego, CA). All of the data were presented as the mean±standard error of the mean (SEM). The differences were evaluated with analysis of variance followed by the Student–Newman–Keuls test for multiple comparisons and the Student *t* test for pairwise comparisons. A value of p<0.05 was considered to be a statistically significant difference.

## Results

### Semaphorin 3A inhibits vascular endothelial growth factor-165–induced human umbilical vein endothelial cell proliferation

**Figure 1:**
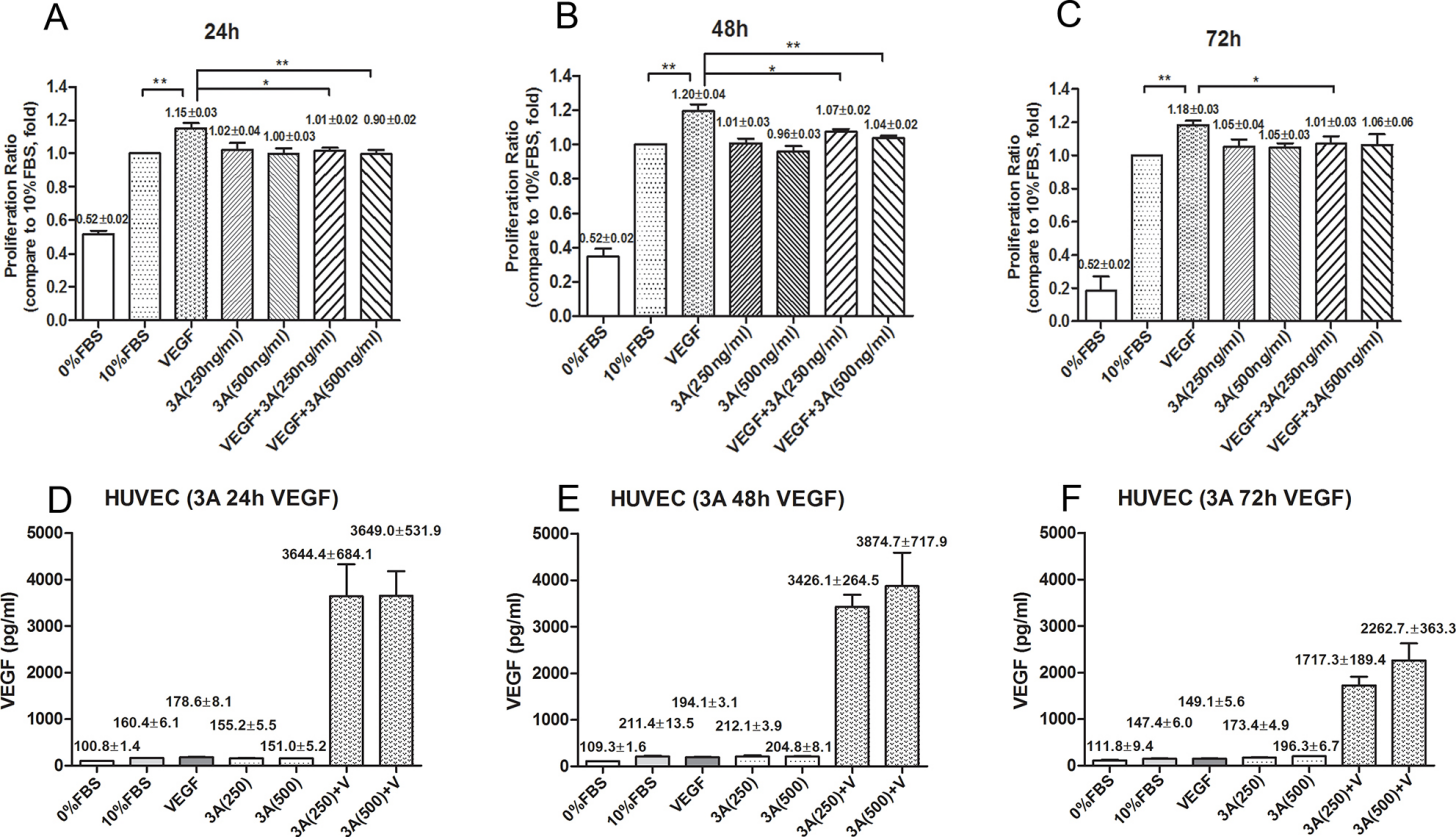
Effects of Sema3A on proliferation and vascular endothelial growth factor (VEGF) secretion of human umbilical vein endothelial cells (HUVECs). **A**: The panel is the statistical results for HUVECs proliferation at 24 h time point. **B**: The panel is the statistical results for HUVECs proliferation at 48 h time point. **C**: The panel is the statistical results for HUVECs proliferation at 72 h time point. In panel **A**, **B** and **C**, the y-axis represents the proliferation ratio that different treatment groups comparing to 10% FBS treatment group, and each experiments were repeated at least three times. **D**: The panel is the statistical results for free VEGF165 secretion as measured by an enzyme-linked immuno sorbent assay (ELISA) at 24 h time point. **E**: The panel is the statistical results for free VEGF165 secretion as measured by an ELISA assay at 48 h time point. **F**: The panel is the statistical results for free VEGF165 secretion as measured by an ELISA assay at 72 h time point. In panel **D**, **E** and **F**, the y-axis represents the value the detected by ELISA Kit and each experiment were repeated at least three times. All of the data were presented as mean± standard error of the mean (SEM). *p<0.05; **p<0.01.

Sema3A did not inhibit HUVEC proliferation in the general culture medium, even at a high concentration (Sema3A 500 ng/ml; compared to the 10% FBS group), but it did inhibit VEGF_165_-induced HUVECs proliferation significantly at both concentrations and at variable time points when compared to the VEGF_165_-treated group (p<0.05; Figure 1A-C).

### Effects of semaphorin 3A on vascular endothelial growth factor-165 secretion

There was no increase in free VEGF_165_ secretion in the Sema3A-treated HUVEC groups (Figure 1D-F, the columns of 3A 250 ng/ml and 3A 500 ng/ml groups) under the general culture condition; on the other hand, under the VEGF_165_ (25 ng/ml) stimulation condition, the detected free VEGF_165_ was much higher in the Sema3A-treated HUVEC (Figure 1D-F, the columns of VEGF+3A 250 ng/ml and VEGF+3A 500 ng/ml groups; p<0.05). The above results indicate that Sema3A did not affect the VEGF_165_ secretion in HUVECs under the general culture condition, but rather inhibited the utilization ability of exogenous VEGF_165_ and impeded its functions. These results can explain the proliferation assay results showing that Sema3A only inhibits VEGF_165_-induced HUVEC proliferation.

### Semaphorin 3A inhibits human umbilical vein endothelial cell migration

**Figure 2:**
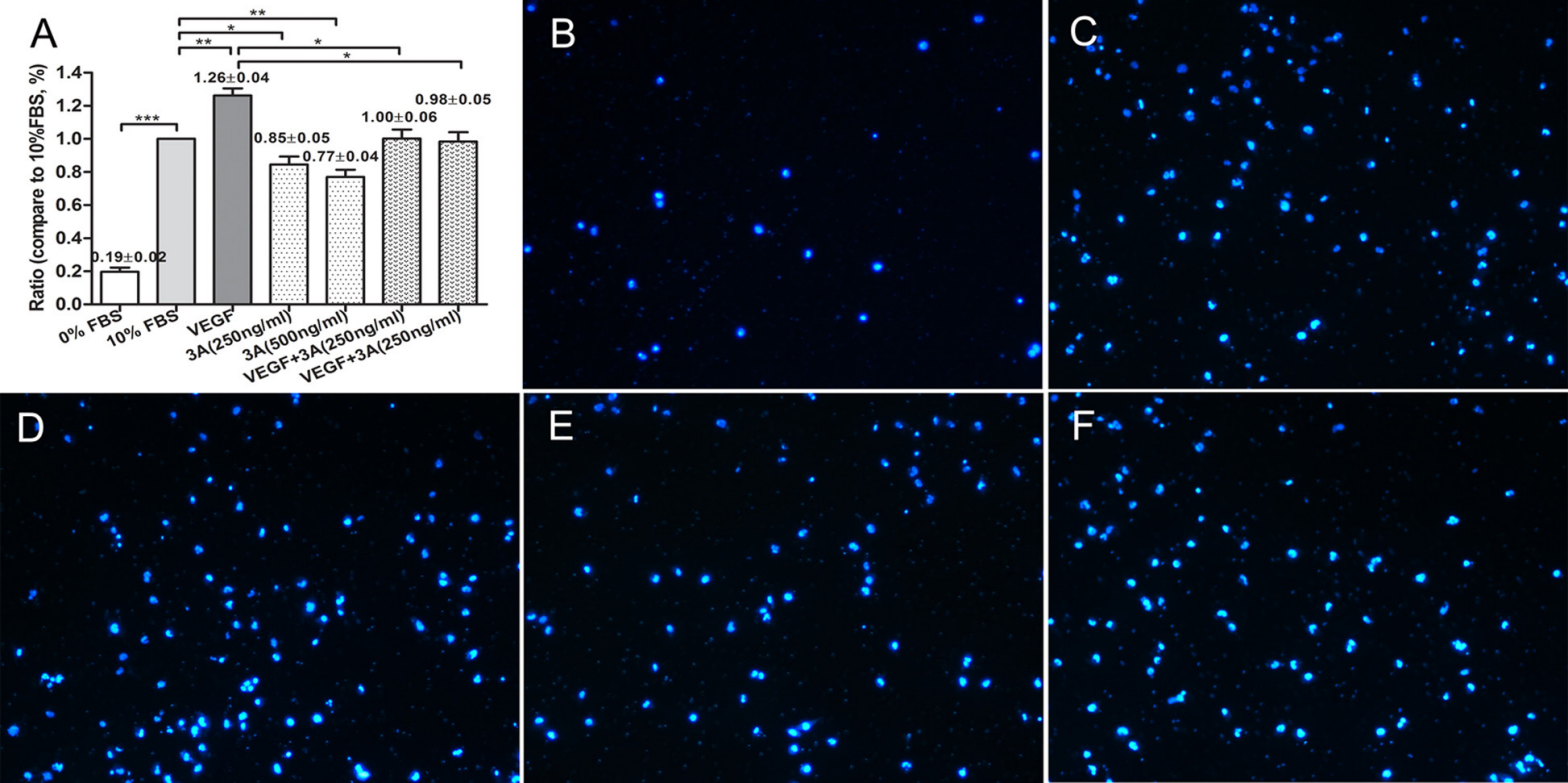
Effects of semaphorin 3A on the migration of human umbilical vein endothelial cells. **A**: The panel is statistical analysis results, y-axis represents the migration ratio of the different treatment groups comparing to the 10% fetal bovine serum (FBS) treatment group. **B**: The panel is 0% FBS-treated group; **C**: The panel is 10% FBS culture group. **D**: The panel is vascular endothelial growth factor-165 (VEGF_165_)-treated group: **E**: The panel is semaphorin 3A (Sema3A) 500ng/ml-treated group. **F**: The panel is Sema3A and VEGF_165_-treated group. In panels **B** to **F**, the cell nuclei were stained with 4',6-diamidine-2'-phenylindole dihydrochloride (DAPI), which is shown as blue dots. All of the pictures (panel **B** to **F**) were taken as the magnificent of 10× by Zeiss florescence microscopy. All of the data were repeated at least three times, and were presented as mean± standard error of the mean (SEM). *p<0.05; **p<0.01; ***p<0.001.

The migration study was assessed with a Transwell assay. As shown in Figure 2, the number of cells that passed through the membrane in the Sema3A-treated HUVEC groups was significantly lower than was the number in the control groups at both concentrations (Figure 2A,C,F, p<0.05). In the VEGF_165_-stimulated group, Sema3A was also able to inhibit the crossing of the HUVECs (Figure 2A,D,E, p<0.05).

### Semaphorin 3A inhibits human umbilical vein endothelial cell tube formation

**Figure 3:**
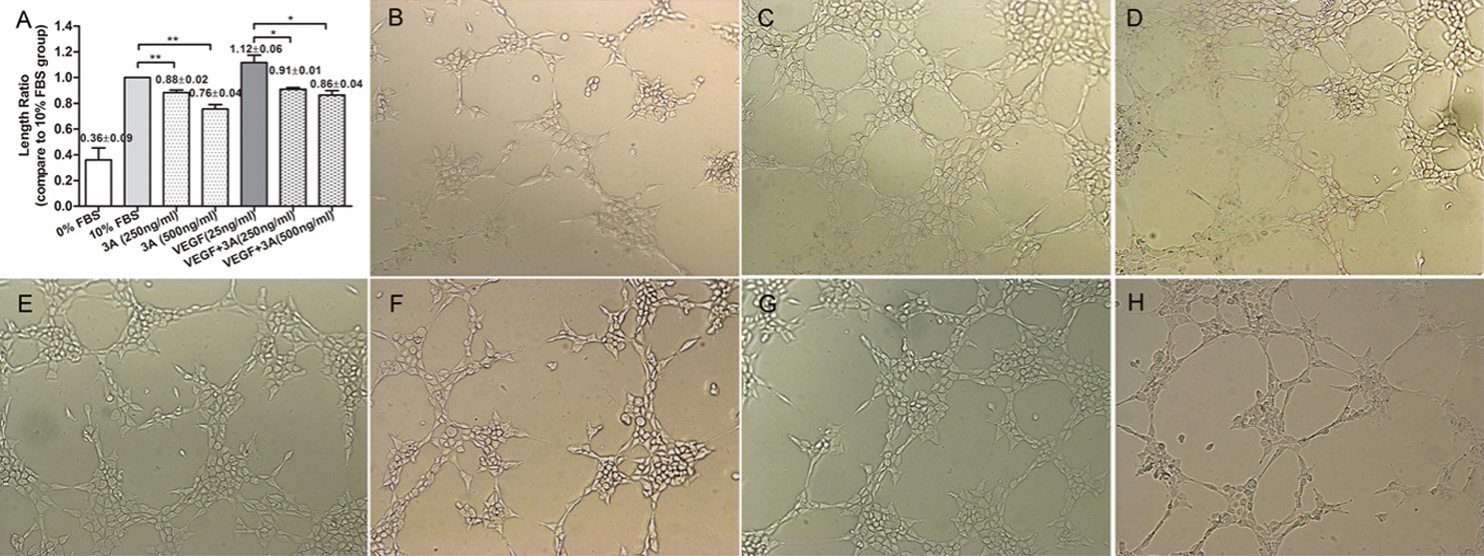
Effects of semaphorin 3A on human umbilical vein endothelial cell tube formation. **A**: The panel shows the statistical analysis results, y-axis represents the length ratio comparing to control group; **B**: The panel is 0% fetal bovine serum (FBS)-treated group; **C**: The panel is 10% FBS culture group; **D**: The panel is vascular endothelial growth factor-165 (VEGF_165_)-treated group; **E**: The panel is semaphorin 3A (Sema3A) 250 ng/ml-treated group; **F**: The panel is Sema3A 500 ng/ml-treated group; **G**: The panel is Sema3A (250 ng/ml) and VEGF_165_-treated group; **H**: The panel is Sema3A (500 ng/ml) and VEGF165-treated group. All of the pictures (panel **B** to **H**) were taken as the magnificent of 10× by Zeiss light microscopy. All of the data were repeated at least three times, and were presented as mean± standard error of the mean (SEM). *p<0.05; **p<0.01.

The Matrigel assay is one of the most widely used methods to evaluate the angiogenesis ability of ECs in vitro. In our study, Sema3A-treated HUVECs showed an impaired capacity to form a regular network at concentrations of 250 ng/ml and 500 ng/ml (Figure 3), both in the general culture medium and in the VEGF_165_-containing medium. The length of the angiogenesis network also showed a statistically significant difference as compared to the control groups.

### Semaphorin 3A induces cell cycle arrest in human umbilical vein endothelial cells but does not induce apoptosis

**Figure 4:**
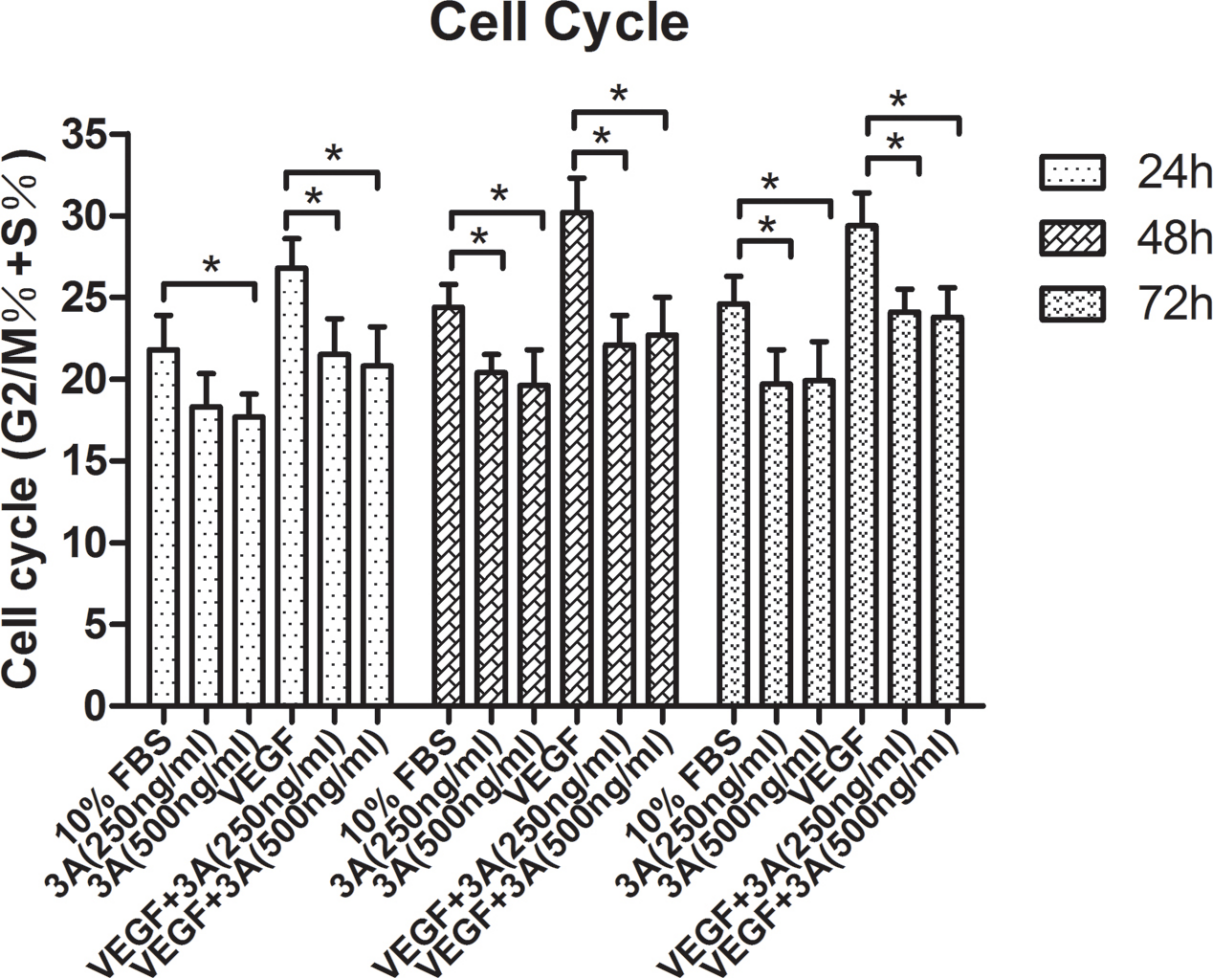
Effects of semaphorin 3A on the cell cycle of human umbilical vein endothelial cells. Shown are the cell cycle data of the different human umbilical vein endothelial cell (HUVEC) groups (G2/M phase + S phase). The data are presented as the mean±standard error of the mean (SEM). Each experiment was repeated at least three separate times. *p<0.05; **p<0.01.

Fluorescence-activated cell sorting was used to evaluate the cell cycle and early and late apoptosis effects. In our study, there was no significant difference between the Sema3A treated groups and the control groups (data not shown) in the apoptosis study. However, Sema3A was able to induce HUVEC cell cycle arrest both in the general culture medium and in the VEGF_165_-induced conditions (Figure 4).

### Western blot analysis of the JNK and p38MAPK signaling pathways

**Figure 5:**
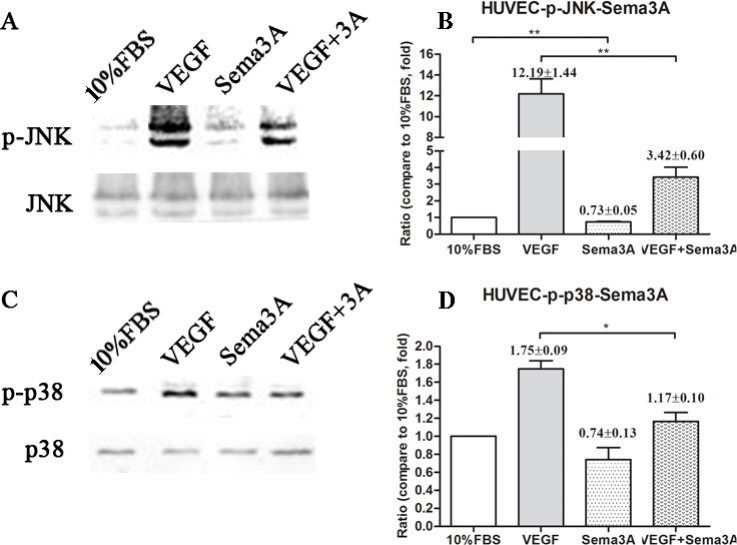
Effects of semaphorin 3A on the c-Jun N-terminal kinase and p38 mitogen-activated protein kinases phosphorylation of human umbilical vein endothelial cells. **A**: Immunoblot images for the c-Jun N-terminal kinase (JNK) and phosphorylation c-Jun N-terminal kinase (p-JNK) signal pathway. **B**: Statistical results for JNK and p-JNK blots. **C**: Immunoblot images for the p38 mitogen-activated protein kinases (p38 MAPK), and phosphorylation p38 mitogen-activated protein kinases (p-p38 MAPK) signaling pathways. **D**: Statistical results for p38 MAPK and p- p38 MAPK blots. All of the data are presented as the mean± standard error of the mean (SEM). Each experiment was repeated three separate times. *p<0.05; **p<0.01.

The immunoblot analysis of the JNK, p-JNK, p38MAPK, and p-p38MAPK signaling pathways revealed that Sema3A inhibited the phosphorylation of JNK both in the general culture medium and in the VEGF-containing culture medium (Figure 5A,B); Sema3A also inhibited the phosphorylation of p38MAPK in the VEGF-containing culture medium (Figure 5C,D).

### Semaphorin 3A protects against oxygen-induced retinopathy retinal pathological angiogenesis while not affecting retina vascular endothelial growth factor-165 secretion

**Figure 6:**
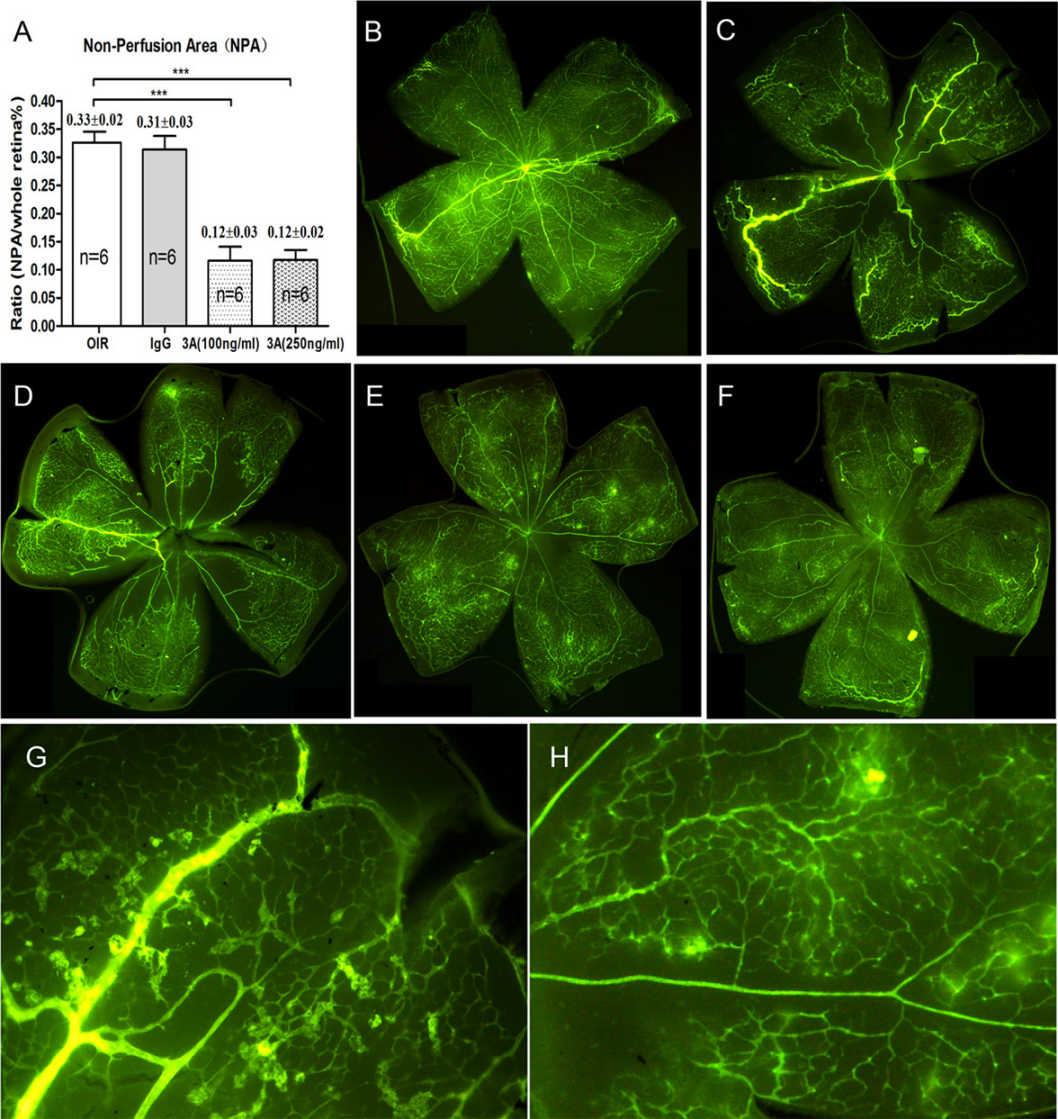
Effects of semaphorin 3A on an oxygen-induced retinopathy model. **A**: Results of statistical analysis; **B**: normal control retina; **C**: oxygen-induced retinopathy (OIR) mouse retina; **D**: immunoglobulin G (IgG)-treated OIR mouse retina; **E**: semaphorin 3A Sema3A (100 ng/ml)-treated retina; **F**: Sema3A (250 ng/ml)-treated retina; **G**: enlarged image (10×) of the top-right part of panel **B**; **H**: enlarged image (10×) of the top-right part of panel **E**. The ratio of the nonperfusion areas (NPAs) is determined by the NPA (the central dark area) as compared to the whole retina *100%, and the y-axis represents the ratios of different groups comparing to OIR untreated group. ***p<0.001.

**Figure 7:**
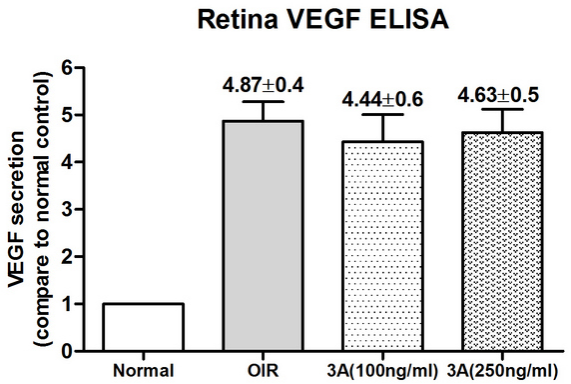
Effects of semaphorin 3A on retina vascular endothelial growth factor-165 (VEGF_165_) secretion. This figure shows the VEGF_165_ in the oxygen-induced retinopathy (OIR) animal model (n=4) and the semaphorin 3A (Sema3A)-treated OIR model (n=4). The y-axis represents the VEGF165 secretion ratios of the different treatment groups comparing to the 10% fetal bovine serum (FBS) treatment group. Each experiment was repeated three separate times. All of the data are presented as the mean± standard error of the mean (SEM).

Sema3A has been shown to inhibit tumor growth [11]. To determine whether Sema3A had an antiangiogenesis effect in the OIR mouse model, Sema3A (100 ng/ml, 250 ng/ml) and IgG (250 ng/ml) were injected intravitreously into the right eyes of retinopathic mice at P12 and to age-matched normal pups. As shown in Figure 6, Sema3A intravitreous injection significantly reduced the neovascularized areas (Figure 6A-F) and reduced abnormal vessel growth (Figure 6G,H). In the ELISA assay that measured VEGF_165_ in the retina of each treated group, however, there was no significant difference between the untreated OIR and the Sema3A-treated retina (Figure 7).

## Discussion

Recently, the application of anti-VEGF therapies, i.e., ranibizumab (Lucentis, Genentech) and bevacizumab (Avastin, Genentech), has played an important role in the treatment of neovascular eye diseases such as ROP, AMD, and central retinal vein occlusion [12]. With the extensive application of these treatments, however, local and systematic side effects appear [13,14], and the limitation of anti-VEGF in retinal neovascularization diseases has aroused the attention of ophthalmologists. Previous studies have stated that intravitreal anti-VEGF treatment causes increased fibrosis in proliferative diabetic retinopathy patients and AMD patients, which is due to the imbalance of VEGF with regard to other growth factors such as connective tissue growth factor [4,15,16]. The unexpected fibrosis tissue will result in membrane contraction, causing retina hole formation and other complications. Therefore, there is a great need to explore and evaluate new antineovascularization compounds or to find supplemental treatment strategies beyond anti-VEGF. As an endogenous antiangiogenesis agent, Sema3A has been studied in tumor angiogenesis and metastasis for years, but its effects on retinal neovascularization are still unclear [17,18].

To our knowledge, this is the first experimental study to address the role of Sema3A in pathological retinal angiogenesis. In the present study, we showed that (a) Sema3A does not affect the secretion of VEGF_165_ either in vitro or in vivo (Figure 1 and Figure 7); (b) Sema3A can inhibit VEGF_165_-induced cell biological effects, such as proliferation, migration, and neovascularization, and its role could involve competing with VEGF_165_ and a possible independent VEGF receptor (VEGFR) effect on HUVECs (Figure 1, Figure 2, Figure 3, and Figure 4); (c) Sema3A can inhibit neovascularization in hypoxia/ischemia-induced retinopathy (Figure 6); (d) the antiangiogenesis effects of Sema 3A could be the results of inhibiting the phosphorylation of the JNK and p38MAPK signaling pathways (Figure 5).

Semaphorins, also known as collapsins, were initially described as axon guidance factors that affect the development of the nervous system [5]. As blood vessels and nerves are structurally similar complex branched systems, the Semas have also been implicated in vessel formation [5]. Class 3 semaphorins (Sema3) represent one of the vertebrate families of semaphorins; they are known to play an important role in tumor biology [17]. In the Sema3 family, Sema3A has been shown to have antiangiogenic properties, and numerous studies have suggested that Sema3A inhibits ECs proliferation, migration, and survival, although the exact mechanism remains unclear [11,16]. In our study, we showed that Sema3A inhibits VEGF_165_-induced HUVEC proliferation, but not in a general culture medium. This inhibition occurs because Sema3A inhibits the utilization of VEGF_165_ (based on our ELISA results in both HUVECs and retinas), and the mechanisms of this inhibition likely result from Sema3A competing with VEGF_165_ for their binding site on neuropilin receptors [19].

Sema3A and VEGF_165_ share a common coreceptor, specifically Nrp-1 [20]. VEGF family members mediate their downstream effects by binding to neuropilins and forming complexes with VEGFRs, which are analogous to the Sema3-neuropilin-Plxn complex [17]. While Nrp-1 is not required for VEGF function, it can enhance the signaling of VEGF through one of its receptor tyrosine kinases, VEGFR2 [16]. This is evidenced by our western blot analysis showing that the VEGFR2 downstream signalings of JNK and p38MAPK phosphorylation were downregulated. In addition to the antiproliferation effect, Sema3A inhibits HUVEC migration and tube formation, both with and without VEGF_165_. These results are consistent with previous studies that showed that Sema3A impairs EC adhesion and migration by negatively regulating the integrin-mediated adhesion of cultured ECs and experimental angiogenesis in vitro and in vivo, as well as inducing the disappearance of EC focal contacts, which is followed by the collapse of the actin cytoskeleton [7,21-23]. Thus, we supposed that besides a simple binding competition between Sema3A and VEGF_165_, there should be other, independent VEGFR effects on HUVEC functions. Although a previous study showed that Sema3A promoted the apoptosis of ECs [24], we did not replicate these results in our study, and we only found that Sema3A induced cell cycle arrest. This result might have been observed because we used immortalized cell lines, while the other studies used primary ECs [24]. Sema3A’s induction of cell cycle arrest in a general culture condition (Figure 4) contradicts the results wherein Sema3A did not affect HUVEC proliferation (Figure 1). The reasons for this may have to do with the sensitivity of our detection method (Cell Counting Kit-8) or that Sema3A did not influence intracellular dehydrogenase. Further mechanisms need to be tested and discussed in future studies.

In the in vivo study, we used a broadly accepted hypoxia/ischemic retinopathy animal model, an OIR mouse model with a mechanism similar to that of ROP [25,26]. In this study, we also showed that Sema3A intravitreal injection in normal mice did not induce changes in the VEGF_165_ expression (Figure 7). Even in the OIR model, Sema3A did not affect the secretion of VEGF_165_, but it was able to reduce the non-perfusion area and the pathological angiogenesis (Figure 6). Another explanation for this result is that Sema3A can compete with VEGF_165_ for binding to its receptor, ultimately impeding the function of VEGF_165_.

In summary, we comprehensively studied the antiangiogenesis effects of Sema3A both in vitro and in vivo, and found that Sema3A could effectively inhibit the growth of retinal neovascularization in an OIR model. Our data suggested that Sema3A may be an innovative approach for future therapeutic strategies against other types of angiogenesis as well. Sema3A could be an adjunctive therapeutic strategy for VEGF_165_ inhibitors. To our knowledge, this study provides the first evidence of the anti–retinal neovascularization potential of Sema3A.
